# Idiopathic and isolated adrenocorticotropic hormone deficiency presenting as continuous epigastric discomfort without symptoms of hypoglycemia: a case report

**DOI:** 10.1186/s13256-019-2050-7

**Published:** 2019-04-30

**Authors:** Seizo Okauchi, Fuminori Tatsumi, Yuki Kan, Megumi Horiya, Akiko Mizoguchi, Yoshiro Fushimi, Junpei Sanada, Momoyo Nishioka, Masashi Shimoda, Kenji Kohara, Shuhei Nakanishi, Kohei Kaku, Tomoatsu Mune, Hideaki Kaneto

**Affiliations:** Department of Diabetes, Endocrinology and Metabolism, Kawasaki Medical School, 577 Matsushima, Kurashiki, 701-0192 Japan

**Keywords:** ACTH deficiency, Epigastric discomfort, General malaise, Hypoglycemia, Case report

## Abstract

**Background:**

Isolated adrenocorticotropic hormone deficiency is one kind of hypopituitarism and is triggered by various diseases including autoimmune disorder and/or autoimmune hypophysitis. Adrenocorticotropic hormone deficiency brings out various serious symptoms such as severe hypoglycemia, hypotensive shock, and disturbance of consciousness.

**Case presentation:**

Here we report a case of 65-year-old Japanese man who developed idiopathic and isolated adrenocorticotropic hormone deficiency. He had continued epigastric comfort without any symptom of hypoglycemia or any autoimmune abnormality. Since he continued to complain of mild epigastric discomfort and general malaise, he was misdiagnosed as having functional dyspepsia and a depression state and took medicine for them for several months. Infection markers and several antibodies which we examined were all negative. An abdominal computed tomography scan showed no mass in adrenal tissue; contrast magnetic resonance imaging of his brain showed that pituitary size was within normal range, and pituitary gland deep dyeing delay and/or deeply stained deficit were not observed. However, in a corticotropin-releasing hormone load test, response of adrenocorticotropic hormone and cortisol was poor after corticotropin-releasing hormone loading, and in growth hormone-releasing peptide 2 load test, adrenocorticotropic hormone response was poor, suggesting the presence of adrenocorticotropic hormone deficiency. Therefore, we started treatment with hydrocortisone, and his various symptoms were soon mitigated.

**Conclusions:**

We should bear in mind the possibility of adrenocorticotropic hormone deficiency even when patients complain of epigastric discomfort or general malaise alone.

## Introduction

Isolated adrenocorticotropic hormone (ACTH) deficiency is one kind of hypopituitarism and it is triggered by various diseases including autoimmune disorder and/or autoimmune hypophysitis [1–5]. In fact, anti-pituitary antibody is often observed, and various autoimmune disorders, such as type 1 diabetes and Crohn’s disease, often accompany ACTH deficiency. Therefore, the presence of autoimmune abnormality helps us to diagnose the disease.

Isolated ACTH deficiency brings out various serious symptoms such as severe hypoglycemia, hypotensive shock, and disturbance of consciousness. Hypopituitarism usually brings out adrenal deficiency which is often accompanied by similar symptoms. In clinical practice, such serious symptoms, including severe hypoglycemia, hypotensive shock, and disturbance of consciousness, could help us to diagnose hypopituitarism and/or adrenal deficiency.

Here we report a case of 65-year-old man who developed idiopathic and isolated ACTH deficiency without any symptom of hypoglycemia or any autoimmune abnormality.

## Case presentation

In March 2017, a 65-year-old Japanese man, a ship designer, had mild epigastric discomfort and general malaise. An attending doctor thought that he had a digestive tract disease; in upper gastrointestinal endoscopy, however, there was no abnormality. He continued to complain of epigastric comfort and general malaise; he was misdiagnosed as having functional dyspepsia and depressive state, and he started taking medicine for them. He continued the same treatment for approximately 6 months, but the symptoms did not disappear. In September, 2017, he had nausea and vomiting, and finally he could not take any meal. He was then hospitalized in our institution so that we could supply him with nutrition.

His height and body weight were 169 cm and 52.9 kg. Systolic and diastolic blood pressure and heart rate were 119/87 mmHg and 87 beats/minute. Body temperature was increased up to 38.5 °C. In physical examination, there was no special abnormality in his heart, lungs, and abdomen. Table 1 shows the clinical characteristics on admission. His C-reactive protein (CRP) was increased up to 13.36 mg/dL, suggesting the presence of inflammation. An increase of blood urea nitrogen (BUN) and uric acid was observed which we think was probably due to dehydration. Although he had high fever and high CRP, all the infection markers that we examined were negative. In addition, several antibodies which we examined were all negative. Taking into account these data, we thought it unlikely that he had some inflammatory disease and/or autoimmune disorder such as collagen disease. Since his blood glucose level was relatively low and the number of eosinophils was relatively high, we examined the possibility of adrenal deficiency. As shown in Table 1, ACTH and cortisol levels were low and urinary cortisol level was also low, suggesting the presence of ACTH deficiency and adrenal insufficiency. An increased prolactin level was also observed which we assumed was induced by the side effect of dopamine blockers.

**Table 1:** Laboratory data

**Peripheral blood**		**Diabetes and endocrine markers**		**Infection markers**	
RBC	408 × 10^4^/μL	HbA1c	4.8%	HBs Ag	(−)
Hemoglobin	12.5 g/dL	Plasma glucose	56 mg/dL	HCV Ab	(−)
Hematocrit	36.9%	TSH	5.37 μU/mL	HIV	(−)
WBC	10,460/μL	FT3	3.38 pg/mL	TPHA	(−)
Neut	45.6%	FT4	1.06 ng/dL	β-D-glucan	< 6 pg/mL
Eo	6.7%	ACTH	1.6 μU/mL	TB Ab	< 0.05 U/mL
Baso	0.7%	Cortisol	0.5 μg/dL	QFT	(−)
Mono	11.1%	DHEA-S	32 μg/dL	CMV-C7-HRP	(−)
Lymph	35.9%	GH	2.94 ng/dL	**Immune response markers**	
Platelet	17.1 × 10^4^/μL	IGF-1	51 ng/mL	ANA	(−)
**Blood biochemistry**		LH	4.32 mU/mL	RF	(−)
Total protein	6.9 g/dL	FSH	4.96 mU/mL	Scl-70 Ab	(−)
Albumin	3.6 g/dL	Prolactin	66.4 ng/mL	ACA	(−)
Total bilirubin	1.2 mg/dL	Renin activity	0.2 ng/mL/hr	Tg Ab	< 10.0 U/mL
AST	33 U/L	Aldosterone	34.3 pg/mL	TPO Ab	9.1 U/mL
ALT	22 U/L	Adrenaline	58 pg/mL	Anti-pituitary Ab	(−)
LDH	146 U/L	Noradrenaline	1882 pg/mL	IgG4	78.7 mg/dL
ALP	163 U/L	Dopamine	63 pg/mL	**Tumor marker**	
γ-GTP	21 U/L	Urinary cortisol	≤10.3 μg/day	CEA	1.2 ng/dL
Creatinine	0.82 mg/dL	**Electrolytes**		CA19–9	9.5 U/mL
BUN	29 mg/dL	Na	134 mEq/L	PSA	0.7 ng/mL
Uric acid	9.0 mg/dL	K	4.5 mEq/L	**Urine**	
Amylase	71 U/L	Cl	98 mEq/L	Sugar	(−)
CRP	13.36 mg/dL	Ca	10.1 mg/dL	Protein	(−)
BNP	49.5 pg/mL	IP	4.3 mg/dL	Ketone body	(3+)

*Ab* antibody, *ACA* anticentromere antibody, *ACTH* adrenocorticotropic hormone, *Ag* antigen, *ALP* alkaline phosphatase, *ALT* alanine aminotransferase, *ANA* antinuclear antibodies, *AST* aspartate aminotransferase, *Baso* basophils, *BNP* brain natriuretic peptide, *BUN* blood urea nitrogen, *CA19-9* cancer antigen 19-9, *CEA* carcinoembryonic antigen, *CMV* cytomegalovirus, *CRP* C-reactive protein, *DHEA-S* dehydroepiandrosterone sulfate, *Eo* eosinophil, *FSH* follicle-stimulating hormone, *FT3* free triiodothyronine, *FT4* free thyroxine, *γ-GTP* gamma-glutamyl transpeptidase, *GH* growth hormone, *HbA1c* glycated hemoglobin, *HBs Ag* hepatitis B surface antigen, *HCV* hepatitis C virus, *IGF-1* insulin-like growth factor, *LDH* lactate dehydrogenase, *LH* luteinizing hormone, *Lymph* lymphocytes, *Mono* monocytes, *Neut* neutrophils, *PSA* prostate-specific antigen, *QFT* QuantiFERON, RBC red blood cells, *RF* rheumatoid factor, *TB Ab* tuberculosis antibody, *Tg Ab* thyroglobulin antibody, *TPHA Treponema pallidum* hemagglutination, *TPO Ab* anti-thyroid peroxidase antibody, *TSH* thyroid-stimulating hormone, *WBC* white blood cells

**Fig. 1:**
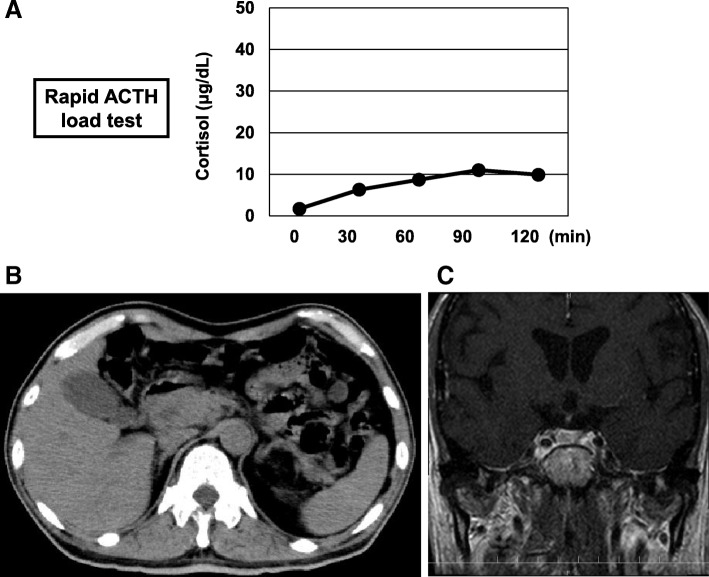
Various image inspections and load tests. **a** Rapid adrenocorticotropic hormone load test. Cortisol level is increased to over 5 μg/dL 60 minutes after the load, but the peak of cortisol is low (11 μg/dL). **b** Abdominal computed tomography scan. There is no mass in adrenal tissue. **c** Brain contrast magnetic resonance imaging. Pituitary size is within normal range, and there is no pituitary gland deep dyeing delay and no deeply stained deficit. *ACTH* adrenocorticotropic hormone

**Fig. 2:**
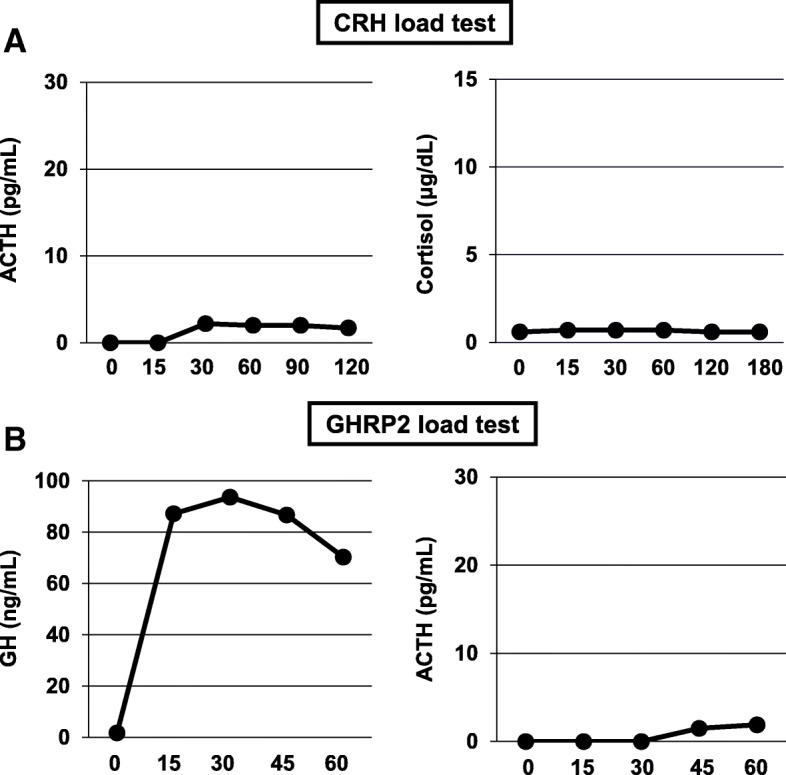
Corticotropin-releasing hormone and growth hormone-releasing peptide 2 load test. **a** Corticotropin-releasing hormone load test. Response of adrenocorticotropic hormone and cortisol is poor after corticotropin-releasing hormone loading. **b** Growth hormone-releasing peptide 2 load test. Adrenocorticotropic hormone response is poor although growth hormone response is preserved. *ACTH* adrenocorticotropic hormone, *CRH* corticotropin-releasing hormone, *GH* growth hormone, *GHRP2* growth hormone-releasing peptide 2

**Fig. 3:**
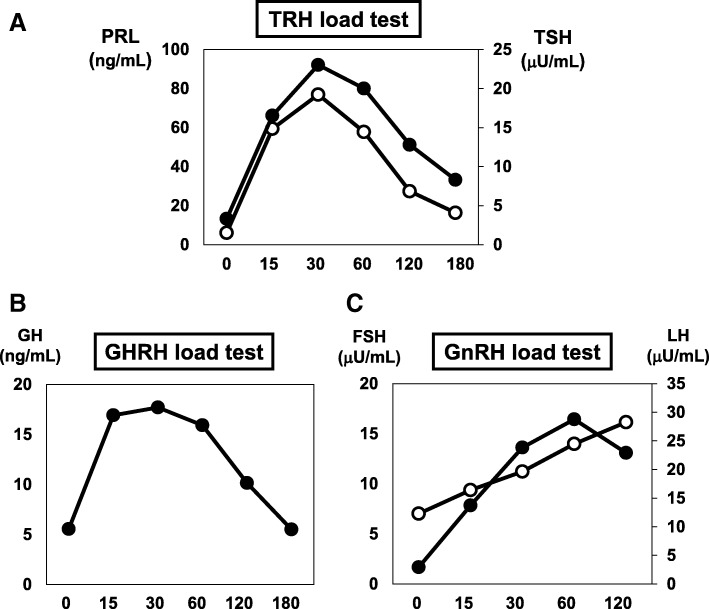
Triple load test: thyrotropin-releasing hormone, growth hormone-releasing hormone, and gonadotropin-releasing hormone load. **a** Thyrotropin-releasing hormone load test. Thyroid-stimulating hormone and prolactin levels are increased after thyrotropin-releasing hormone loading. **b** Growth hormone-releasing hormone load test. Growth hormone level is increased after growth hormone-releasing hormone loading. **c** Gonadotropin-releasing hormone load test. Luteinizing hormone and follicle-stimulating hormone levels are increased after gonadotropin-releasing hormone loading. *FSH* follicle-stimulating hormone, *GH* growth hormone, *GHRH* growth hormone-releasing hormone, *GnRH* gonadotropin-releasing hormone, *LH* luteinizing hormone, *PRL* prolactin, *TRH* thyrotropin-releasing hormone, *TSH* thyroid-stimulating hormone

Next, we performed rapid ACTH load test. As shown in Fig. 1a, his cortisol level was increased to over 5 μg/dL 60 minutes after the load, but the peak of cortisol was not so high (11 μg/dL). In abdominal computed tomography (CT), there was no mass in adrenal tissue (Fig. 1b); in brain contrast magnetic resonance imaging (MRI), pituitary size was within normal range, and pituitary gland deep dyeing delay and/or deeply stained deficit were not observed (Fig. 1c). As shown in Fig. 2a, in a corticotropin-releasing hormone (CRH) load test, the response of ACTH and cortisol was poor after CRH loading, suggesting the presence of ACTH deficiency. In addition, in a growth hormone-releasing peptide 2 (GHRP2) load test, ACTH response was poor although growth hormone (GH) response was preserved (Fig. 2b). Next, we performed a triple load test: thyrotropin-releasing hormone (TRH), GH-releasing hormone (GHRH), and gonadotropin-releasing hormone (GnRH) load. As shown in Fig. 3a, in a TRH load test, thyroid-stimulating hormone (TSH) and prolactin levels were increased after TRH loading. In a GHRH load test, GH level was increased after GHRH loading (Fig. 3b). In a GnRH load test, luteinizing hormone (LH) and follicle-stimulating hormone (FSH) levels were increased after GnRH loading. These data suggest that our patient had isolated ACTH deficiency. Since there was no abnormality in brain MRI and in various markers for autoimmune and/or infection diseases, we diagnosed him as having idiopathic and isolated ACTH deficiency.

**Fig. 4:**
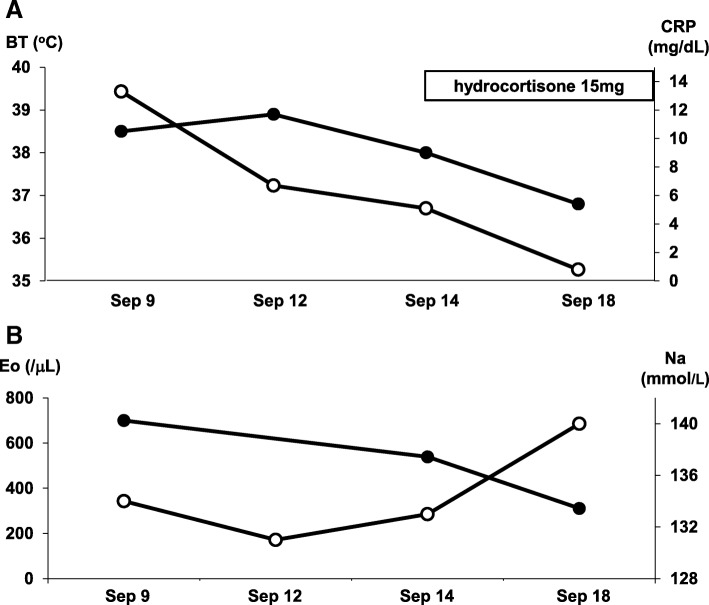
Time course of various clinical parameters. **a** Time course of body temperature and C-reactive protein levels. After starting the treatment with hydrocortisone, body temperature and C-reactive protein levels are decreased. **b** Time course of sodium and eosinophil levels. After starting the treatment with hydrocortisone, sodium level is increased and eosinophil level is decreased after the treatment. *BT* body temperature, *CRP* C-reactive protein, *Eo* eosinophil, *Na* sodium

After diagnosis of isolated ACTH deficiency, we started hydrocortisone on September 14, 2017. As shown in Fig. 4a, after starting the treatment with hydrocortisone, his body temperature and CRP were decreased. In addition, his sodium level was gradually increased and eosinophil level was gradually decreased after the treatment (Fig. 4b). Various symptoms such as nausea, vomiting, appetite loss, and general malaise were mitigated soon after the treatment.

## Discussion

In this case report, we described a patient with idiopathic and isolated ACTH deficiency who had continuous epigastric discomfort without any specific and/or serious symptoms such as severe hypoglycemia, hypotensive shock, and disturbance of consciousness. Therefore, we should consider the possibility of pituitary and/or adrenal diseases even when patients complain of epigastric discomfort or general malaise alone. In addition, our patient’s blood glucose level was relatively low and his number of eosinophils was relatively high. These data suggest that we should bear in mind the possibility of adrenal failure even when patients have mild hypoglycemia and/or mild eosinophilia.

It has been reported that adult ACTH deficiency is often induced by an autoimmune disorder and/or autoimmune hypophysitis and the presence of autoimmune abnormality often helps us to diagnose the disease in clinical practice [1–5]. However, the patient in this case report indicates that ACTH deficiency is not necessarily accompanied by autoimmune abnormality. In patients without autoimmune abnormality, such as the present patient, it is quite difficult to diagnose ACTH deficiency. Therefore, we should be careful not to do misdiagnose ACTH deficiency as some functional gastrointestinal disease or mental disorder.

There is a limitation in this case report; for example, ACTH deficiency may be symptomatic in several ways and epigastric discomfort is a less common but a known symptom. In this way, the case might represent a limited contribution to medical research. However, we believe that this case report will help clinicians to diagnose ACTH deficiency.

Taken together, we should bear in mind the possibility of ACTH deficiency even when patients complain of epigastric discomfort or general malaise alone.

## Conclusions

We should bear in mind the possibility of idiopathic and isolated ACTH deficiency when patients complain of mild but continued epigastric discomfort and/or general malaise even without any symptom of hypoglycemia or any autoimmune abnormality.
